# Metformin represses the carcinogenesis potentially induced by 50 Hz magnetic fields in aged mouse fibroblasts via inhibition of NF‐kB

**DOI:** 10.1111/jcmm.70132

**Published:** 2024-10-01

**Authors:** Tugba Soydas, Guven Yenmis, Matem Tuncdemir, Mustafa Tunaya Kalkan, Elif Yaprak Sarac, Ayhan Bilir, Gonul Kanigur Sultuybek

**Affiliations:** ^1^ Department of Medical Biology, Medical Faculty Istanbul Aydin University Istanbul Turkey; ^2^ Department of Medical Biology Istanbul University‐Cerrahpasa, Cerrahpasa Faculty of Medicine Istanbul Turkey; ^3^ Department of Medical Biology Hatay Mustafa Kemal University, Tayfur Sokmen Faculty of Medicine Hatay Turkey; ^4^ Department of Medical Biophysics Istanbul Aydin University, Medical Faculty Istanbul Turkey; ^5^ Department of Molecular Biology‐Genetics and Biotechnology Istanbul Technical University, Faculty of Science and Letters Istanbul Turkey; ^6^ Department of Histology and Embryology Atlas University, Medical Faculty Istanbul Turkey

**Keywords:** aging, cancer, extremely low ‐frequency magnetic fields, metformin, MMP2/9, NF‐kB

## Abstract

Aging is a risk factor for various human disorders, including cancer. Current literature advocates that the primary principles of aging depend on the endogenous stress‐induced DNA damage caused by reactive oxygen species 50 Hz low‐frequency magnetic field was suggested to induce DNA damage and chromosomal instability. NF‐kB, activated by DNA damage, is upregulated in age‐related cancers and inhibition of NF‐kB results in aging‐related delayed pathologies. Metformin (Met), an NF‐kB inhibitor, significantly reduces both NF‐kB activation and expression in aging and cancer. This in vitro study, therefore, was set out to assess the effects of 5mT MF in 50 Hz frequency and Met treatment on the viability and proliferation of aged mouse NIH/3T3 fibroblasts and expression of *RELA/p65*, matrix metalloproteinases *MMP2* and *MMP9*, and E‐cadherin (*CDH1*) genes. The trypan blue exclusion assay was used to determine cell viability and the BrdU incorporation assay to determine cell proliferation. The MMP‐2/9 protein analysis was carried out by immunocytochemistry, NF‐kB activity by ELISA and the expressions of targeted genes by qRT–PCR methods. Four doses of Met (500 uM, 1 mM, 2 mM and 10 mM) suppressed both the proliferation and viability of fibroblasts exposed to the MF in a dose‐dependent pattern, and the peak inhibition was recorded at the 10 mM dose. Met reduced the expression of *NF‐kB*, and MMP2/9, elevated *CDH1* expression and suppressed NF‐kB activity. These findings suggest that Met treatment suppresses the carcinogenic potential of 50 Hz MFs in aged mouse fibroblasts, possibly through modulation of NF‐kB activation and epithelial‐mesenchymal transition modulation.

## 1 INTRODUCTION

Extremely low‐frequency magnetic fields (ELF‐MF) are generated during the production and transmission of electricity, as seen in power lines, railways and household electrical devices. On a daily basis, we encounter 50–60 Hz ELF‐EMFs emitted by the majority of household electrical appliances.^1^ Numerous epidemiological research findings indicate that exposure to ELF‐EMF has been associated with an increased risk of cancer development, encompassing leukaemia, brain tumours and breast cancers.^2^ In 2002, the International Agency for Research on Cancer (IARC) categorized ELF‐EMFs as a possible carcinogen, group 2B (IARC, 2002). Studies in this field suggest that exposure to low‐intensity and low‐frequency electromagnetic fields may alter DNA integrity, potentially initiating carcinogenic processes or accelerating the development or spread of existing cancers.^3^, ^4^

Brabant et al. identified a correlation between ELF‐MF exposure and an increased risk of childhood leukaemia.^2^ Giorgi and Del Re conducted a comprehensive review of epidemiological studies and found compelling evidence supporting this association. The evidence presented in this review indicates that ELF‐MF exposure may contribute to an elevated risk of childhood leukaemia and a clear link exists between epigenetic alterations and pathological conditions, including cancer, as well as aging.^5^ Furthermore, a meta‐analysis of ELF‐EMF exposure and breast cancer risk in postmenopausal women suggests that ELF‐EMF may lead to augmented risk of breast cancer.^6^

Despite the limited data available from experimental studies, the potential for ELF‐MF exposure to induce adverse health effects remains a subject of ongoing discussion.^7^, ^8^ Although there is a general consensus regarding the negative effects of EMFs, some studies have highlighted the positive effects of magnetic field (MF) therapy, particularly in the cancer treatment, especially when combined with anticancer drugs.^9^, ^10^ Although ELF‐MF exposure alone appears to have a negligible or insignificant effect on apoptosis, its interaction with other factors, such as chemotherapeutic agents or ionizing radiation, can lead to unpredictable and sometimes paradoxical outcomes. This complex interplay likely contributes to the conflicting evidence regarding ELF‐MF's influence on cellular death.^11^ It is hypothesized that ELF‐MF exposure could modify the properties of breast cancer cells and enhance doxorubicin's (a prominent chemotherapeutic agent) anti‐proliferative efficacy.^12^ Ramazi et al. discovered that ELF‐MF exposure increased the effectiveness of doxorubicin by inducing a high level of cell toxicity and stimulating the production of reactive oxygen species (ROS).^13^ Epidemiological cancer studies in humans support these findings, suggesting that ELF‐MF exposure can cause DNA damage, potentially leading to cancer and related diseases.^14^

There are growing concerns about the potential biological hazards associated with ELF‐EMFs. In particular, the MFs at 50–60 Hz frequency had a contributory effect between aging and cancer.^15^, ^16^, ^17^ Falone et al. showed that exposure to ELF‐MF causes a significant weakening of antioxidant defence systems in aged rat brain.^15^ ROS based cellular damage accumulation is the key concept underlying the functional losses associated with aging, which is the basis of the oxidative stress theory.^18^ Recent experimental studies suggest that elevated intracellular ROS levels, owing to an ELF‐MF exposure, thus, enable several cellular modifications including DNA damage, chromosomal instability and apoptosis.^15^, ^16^, ^17^ For instance, a 1 mT EMF at 60 Hz induced chromosomal instability in human fibroblasts and a 5 mT EMF at 60 Hz caused cell death by generating ROS in human HL‐60 promyelocytic leukaemia cells.^16^, ^17^ Additionally, a 14 μT EMF at 60 Hz triggered apoptosis in mouse testicular germ cells, while a 100 μT EMF at 50 Hz halted the cell cycle at the G1 phase in human SH‐SY5Y neuroblastoma cells.^19^, ^20^ Regarding the effects on cellular processes in cancerogenesis, in 2023, a proteomic data analysis conducted by Lazzarini et al. identified changes in MDA‐MB‐231 breast cancer cells exposed to 50 Hz ELF‐MF, demonstrating a decrease in adhesive properties and an increase in cell migration and invasion capabilities.^21^

Constitutive activation of nuclear factor‐kappa B (NF‐kB) has been identified as a hallmark of both aging and cancer cells in various studies, including our previous papers.^22^, ^23^, ^24^, ^25^ NF‐kB/Rel protein is formed by a combination of two different subunits, p50 and p65, respectively. The nuclear translocation and elevated expression of the p65 subunit were reported to be directly correlated with the transcriptional NF‐kB activity.^26^ The participation of constitutive NF‐kB (p65 subunit) activity in the expression of epithelial‐to‐mesenchymal transition (EMT), prometastatic and proangiogenic genes including E‐cadherin and matrix metalloproteinases (MMPs) in cancer cells was confirmed through numerous studies. During EMT, the downregulation of e‐cadherin through a complex network of signalling pathways and transcription factors, including NF‐kB, initiates cancer metastasis.^27^, ^29^ The findings of Kim et al. (2017) strongly suggest that ELF‐EMF exposure can activate the NF‐κB signalling pathway in murine macrophage cells, leading to increased inflammation.^29^ This evidence supports the hypothesis that ELF‐EMF may play a role in inflammatory processes. Moreover, two of the MMPs, MMP‐2 and MMP‐9 have also been attributed to metastatic cancer development and progression through their functions in cell proliferation, angiogenesis and migration.^30^

NF‐κB inhibitors are generally used to reduce and/or prevent cancer metastasis by suppressing the expression of these prometastatic genes.^31^ Metformin (Met), one such inhibitor, has a globally accepted anti‐cancer effect, potentially inducing cellular apoptosis and arresting the cancer cell cycle, thereby suppressing tumour proliferation.^32^ Moiseeva et al. demonstrated that Met exerts its anticancer effects through growth and invasion inhibition by blocking NF‐κB activity and expression. However, the precise mechanisms by which Met protects aged cells exposed to ELF‐MF remain largely unexplored.^33^

This study investigates the potential of Met to mitigate cancer/aging‐related effects induced by 50 Hz MF exposure (5 mT) in aged mouse NIH/3T3 fibroblasts. We will assess cell viability, proliferation, expression of MMPs (MMP‐2 and MMP‐9), E‐cadherin (CDH1), RELA/p65 genes and NF‐kB (p65) DNA binding activity. By elucidating the molecular mechanisms underlying 50 Hz MF‐mediated carcinogenesis in these cells and the protective effects of Met administration, we aim to reveal the versatile functions of Met in the context of viability, proliferation and metastasis of aged mouse NIH/3T3 fibroblasts.

## 2 MATERIALS AND METHODS

### 2.1 Cell culture

The NIH/3T3 mouse fibroblasts were purchased from ATCC. The cells were cultured at 37°C in a 5% CO_2_‐containing incubator using Dulbecco's modified Eagle's medium including 10% fetal bovine serum, 100 U/mL penicillin and 100 μ g/mL streptomycin. The cells in their exponential growth stage were digested by trypsin and passaged into T25 flasks. After the 15th–20th passages, cultured NIH/3T3 fibroblasts were used as an in vitro model for cell aging, as previously mentioned.^23^ The cells were then exposed to four different concentrations of Met HCL‐Wanbury (H10000691) for 24 h: 500 μM, 1 mM, 2 mM and 10 mM. The six experimental groups were as follows: the control group (without Met and without MF), the 50 Hz MF group, and four distinct Met administered groups (50 Hz MF + 500 μM Met group, 50 Hz MF + 1 mM Met group, 50 Hz MF + 2 mM Met group, 50 Hz MF + 10 mM Met group). The cells of Met administered groups were incubated with a medium prepared with four different concentrations of Met (500 μM, 1 mM, 2 mM and 10 mM) for 24 h before the application of 50 Hz MF for 1 h.

### 2.2 MF exposure setup

**FIGURE 1:**
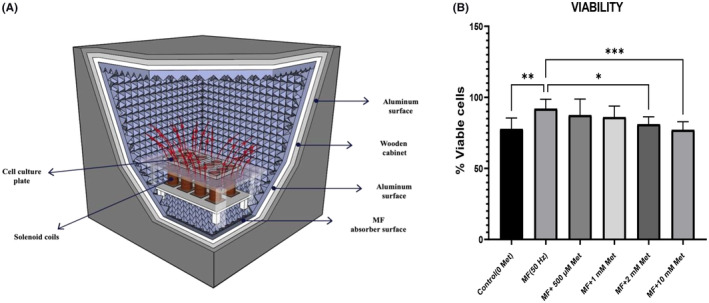
(A) Schematic diagram of the 50 Hz 5 mT MF exposure system for experimental studies. (B) Effects of metformin (500 μM, 1 mM, 2 mM and 10 mM) treatment on viability in aged mouse fibroblast exposed to 50 Hz MF. *p = 0.02; **p = 0.001; ***p = 0.006. Data were expressed as the mean ± SD from five independent experiments.

Eight serially connected copper solenoid coils, each having 560 turns, were used to generate the MF according to previous studies.^34^ The magnetic flux intensity was improved by filling the cores of the coils with soft iron rods and secured with clamps to improve MF strength. The coils were mounted vertically, and the cell culture flasks were kept 1.2 cm above the coils to prevent vibration. Additionally, the coils were isolated from the cell culture flasks using wooden spacers (each 1 cm thick) to prevent heat transfer from the coils to the cell cultures (Figure 1A). The electrical connection was established by connecting the 220 V sinusoidal city electric systems at 50 Hz frequency The experimental set‐up was maintained by stabilizing the system at 90%–95% humidity and 37°C temperature. The MF strength was measured at five different predetermined points in culture flasks and confirmed to be 5 mT using a Leybold Heraeus 54050 Hall effect tesla meter as the applied current passed through the coils.

### 2.3 Cell viability determined by the trypan blue assay

The trypan blue exclusion assay (0.4 percent, Sigma‐Aldrich, Germany) was used to detect the viability of NIH/3T3 fibroblasts. In brief, the centrifuged cell supernatant was resuspended in 1 mL of phosphate‐buffered saline (PBS). An equal volume of trypan blue and the cell suspension were mixed, and the cell‐trypan blue mixture was incubated at room temperature for 3 min. A drop of the suspension was then placed in a haemocytometer and observed under a microscope. The viability of the cells was determined according to their stained or unstained status. The proportion of viable cells (/mL) was estimated using formula of [(total viable cells/ml)/(total cell count/ml)] × 100 after multiplying by two (dilution factor).

### 2.4 Analysis of proliferation by the BrdU incorporation assay

5‐Bromo‐2′‐deoxyuridine (BrdU) was prepared according to the manufacturer's instructions (Sigma‐Aldrich, Schnelldorf, Germany). BrdU was monitored through the visual colorimetric staining procedures mentioned previously in the literature.^26^ A camera‐attached light microscope (CKX41; Olympus, Tokyo, Japan) was used to capture the cell images.

### 2.5 Immunocytochemical analysis of the expression levels of MMP‐2 and MMP‐9

The aged NIH/3T3 cells were resuspended, washed with PBS and eventually fixed with 70% ethanol for 10 min. The fixed cells were then washed twice to prepare them for immunostaining. Briefly, a horseradish peroxidase/AEC Detection IHC Kit (Abcam, USA) was used for the indirect streptavidin‐biotin‐peroxidase reaction. A 1:200 diluted MMP‐2 monoclonal antibody (Invitrogen, USA, MA5‐13590) or MMP‐9 monoclonal antibody (Invitrogen, USA, MA5‐15886) was administered to the cells, followed by incubation overnight at +4°C in the dark. AEC kit (Invitrogen, USA) was used as a chromogen at room temperature, and the cell images were captured under a light microscope. The average number of immuno‐positive cells was calculated by counting the cells at five distinct regions on the samples.

### 2.6 Expression study for *RELA/p65*, *CDH1*, *MMP2* and *MMP9* genes

The aged NIH/3T3 cells that were collected were washed with PBS. RNA isolation was performed using a commercial kit [MasterpureTM RNA Purification (MCR85102, EPICENTRE)]. After spectrophotometric analysis (NanoDrop Eight Spectrophotometer, Thermofisher, USA) to determine the quantity and quality of RNA, the cDNA synthesis and qRT–PCR (Qiagen Quantitect SYBR Green PCR Kit (QIAGEN)) were carried out using self‐designed primer sets (Table 1) using Rotor‐Gene Q (Qiagen, USA). The actin‐Beta gene was used as the housekeeping gene and the expression analysis was held by 2−^∆∆Ct^ calculation.

**TABLE 1:** Primers of genes *RELA/p65*, *MMP2*, *MMP9*, *CDH1 and ACTB* for performing qRT‐PCR.

Gene	Primer	Sequence
*RELA* (*P65*)	Forward	AGTGTGTGAAGAAGCGAGACC
	Reverse	AAATCGGATGTGAGAGGACAG
*MMP2*	Forward	TCATTGGTTACACACCTGACCT
	Reverse	GGGTATCCATCTCCATGCTC
*MMP9*	Forward	TGTCACTTTCCCTTCACCTTC
	Reverse	CTCACTAGGGCAGAAACCAAA
*CDH1*	Forward	CTCCAGTCATAGGGAGCTGTC
	Reverse	CCCAGTCTCGTTTCTGTCTTC
*ACTB*	Forward	ATCTGGCACCACACCTTCTAC
	Reverse	GGTACGACCAGAGGCATACAG

### 2.7 Nuclear extraction preparation of NIH/3T3 cells

Nuclear fractions of the NIH/3T3 fibroblasts were extracted with a commercial nuclear‐cytoplasmic extraction kit (NE‐PER Nuclear and Cytoplasmic Extraction Reagent, Pierce, Rockford, IL, USA) according to the manufacturer's instructions.

### 2.8 Quantification of NF‐κB (p65) DNA‐binding activity

The NF‐kB p65 DNA binding activity was measured using an ELISA‐based method through NF‐kB p65 Transcription Factor assay kit (ab133112; Abcam, UK). In brief, nuclear extracts of NIH/3T3 cells were incubated for 1 h at room temperature on a 96‐well plate pre‐coated with a double‐stranded DNA sequence containing an immobilized NF‐kB response element. NF‐kB, present in nuclear extracts, binds to the NF‐kB response element and is detected using an antibody for NF‐kB/p65 as previously described.^35^ A secondary antibody conjugated to HRP is then added and NF‐kB activation is measured with a plate absorbance reader at 450 nm (Thermo Scientific MULTISKAN GO, Finland).

### 2.9 Statistical analysis

Experiments were repeated five times. Intergroup comparisons were performed by ANOVA and post hoc Tukey's test. The student's *t*‐test was applied to compare two independent groups. GraphPad InStat software (version Prism 8.0.2.) was employed for all statistical analyses. A *p*‐value of less than 0.05 was considered statistically significant.

## 3 RESULTS

### 3.1 The effect of met on the viability of the aged NIH/3T3 exposed to 50 Hz MF

Trypan blue exclusion assay was used to determine the viability of aged NIH/3T3 cells exposed to 50 Hz MF after treatment with four different Met concentrations (500 μM, 1 mM, 2 mM and 10 mM). Cell viability exhibited a dose‐dependent decrease, with the most pronounced inhibition observed at 10 mM Met (*p* < 0.05) (Figure 1B). The results indicate a dose‐dependent decrease in cell viability, with the highest inhibition observed at 10 mM Met, suggesting that high concentrations of Met significantly reduce the viability of aged NIH/3T3 cells under 50 Hz MF exposure.

### 3.2 Met inhibits aged NIH/3T3 cell proliferation induced by 50 Hz MFs

**FIGURE 2:**
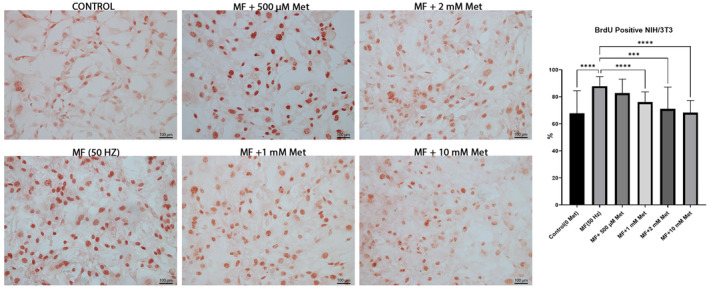
Representative micrographs and statistical analysis of the distribution of anti‐BrdU immunocytochemical staining in aged mouse fibroblasts exposed to 50 Hz MF and in four different dosages of metformin (500 μM, 1 mM, 2 mM and 10 mM). Original magnification, ×40. ***p = 0.0009; ****p < 0.0001. Data were expressed as the mean ± SD from five independent experiments.

The BrdU assay was held to determine the anti‐proliferative action of four doses of Met (500 μM, 1 mM, 2 mM and 10 mM). A 24 h‐incubation with Met blocked the proliferation of the aged NIH/3T3 cells, which have been exposed to 50 Hz MF. When compared to the Met‐free medium, statistically significant inhibition was observed at 10 mM Met concentration (*p* < 0.0001) (Figure 2). The results suggest that 10 mM Met is the most effective dose for inhibiting proliferation in aged mouse fibroblasts exposed to 50 Hz MF.

### 3.3 The effect of Met on the MMP‐2 and MMP‐9 protein expressions of the aged NIH/3T3 exposed to 50 Hz MF

**FIGURE 3:**
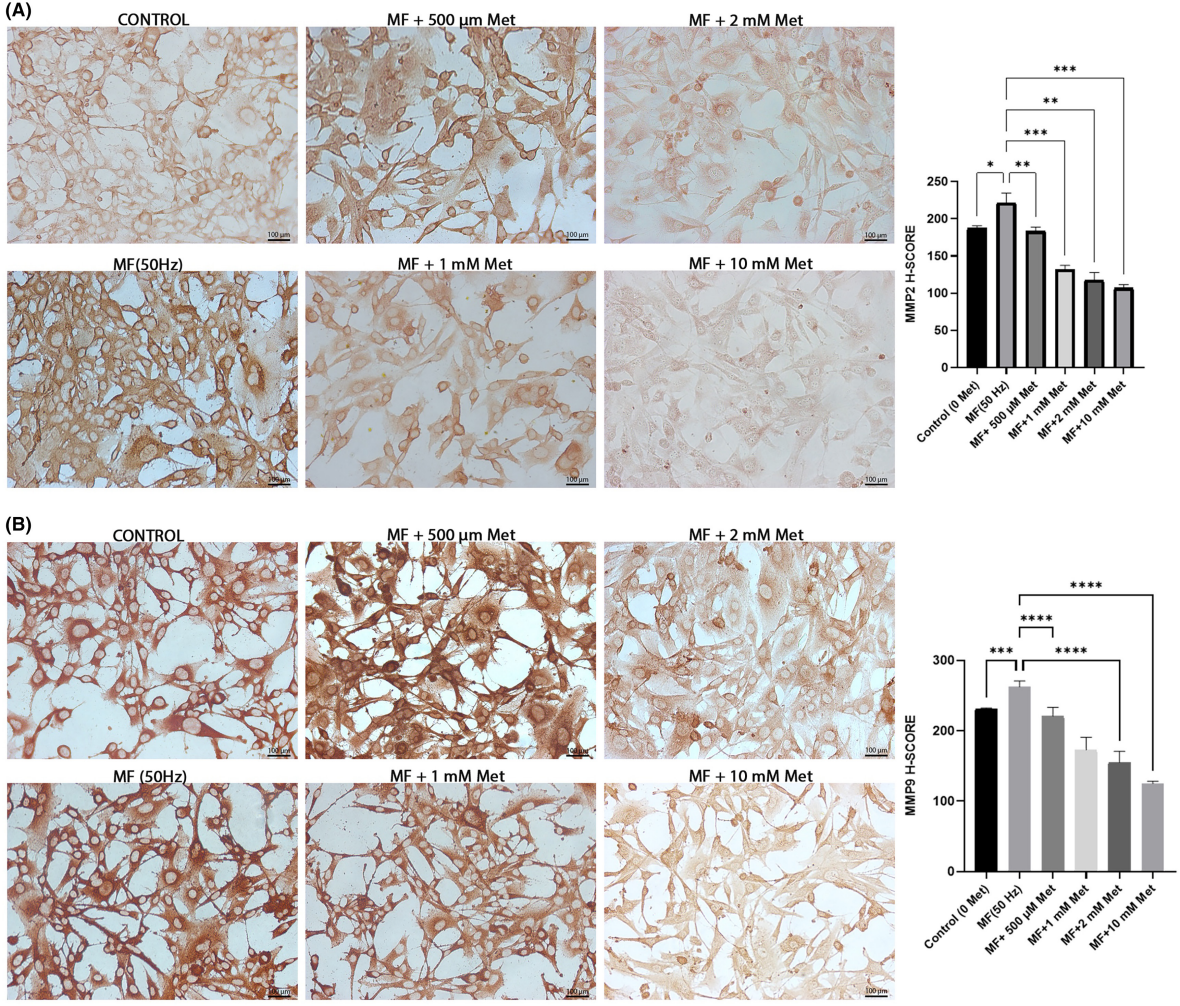
(A) The micrograph images showing the effects of metformin (500 μM, 1 mM, 2 mM and 10 mM) treatment on MMP‐2 protein expressions in aged mouse fibroblast exposed to 50 Hz MF. Original magnification, ×40. *p < 0.05, **p < 0.001; ***p < 0.0001. (B) The micrograph images showing the effects of metformin (500 μM, 1 mM, 2 mM and 10 mM) treatment on MMP‐9 protein expressions in aged mouse fibroblast exposed to 50 Hz MF. Original magnification, ×40, **p < 0.001; ***p < 0.0001. Data were expressed as the mean ± SD from five independent experiments.

The immunoreactivities of MMP‐2 and ‐9 in the aged NIH/3T3 cells exposed to 50 Hz MF were compared between groups with and without Met administration by immunocytochemical staining. The results suggested that MMP‐2 immunopositivity was significantly elevated in the group that was exposed to 50 Hz MF, compared to the control group (*p* = 0.02). A statistically significant decrease in MMP‐2 immunopositivity was observed in the 50 Hz MF groups treated with 1 mM, 2 mM and 10 mM Met concentrations compared to the control group, which was exposed to 50 Hz MF but untreated with Met (Figure 3A).

Similarly, MMP‐9 immunoreactivity was also significantly increased in the NIH/3T3 cell group, which was exposed to 50 Hz MF, compared to the control group (*p* = 0.0009) (Figure 3B). Statistical evaluation of H‐score results (%) of MMP‐9‐positive aged NIH/3T3 cells showed that there was a significant decrease in the 1 mM, 2 mM and 10 mM Met groups compared with the to Met‐free 50 Hz MF group (*p* < 0.001) (Figure 3B). The results also show that a 10 mM concentration of Met is an effective inhibitory dose for MMP 2/9 protein production in aged mouse fibroblasts exposed to 50 Hz MF (*p* < 0.0001). Met treatment, especially at a concentration of 10 mM, significantly reduces the expression of MMP‐2 and MMP‐9 proteins in aged NIH/3T3 cells exposed to 50 Hz MF; this suggests that Met may mitigate the pro‐metastatic effects of MF exposure.

### 3.4 Met regulates RELA (p65), MMP2/9 and CDH1 gene expressions in aged mouse fibroblasts exposed to 50 Hz MFs

**FIGURE 4:**
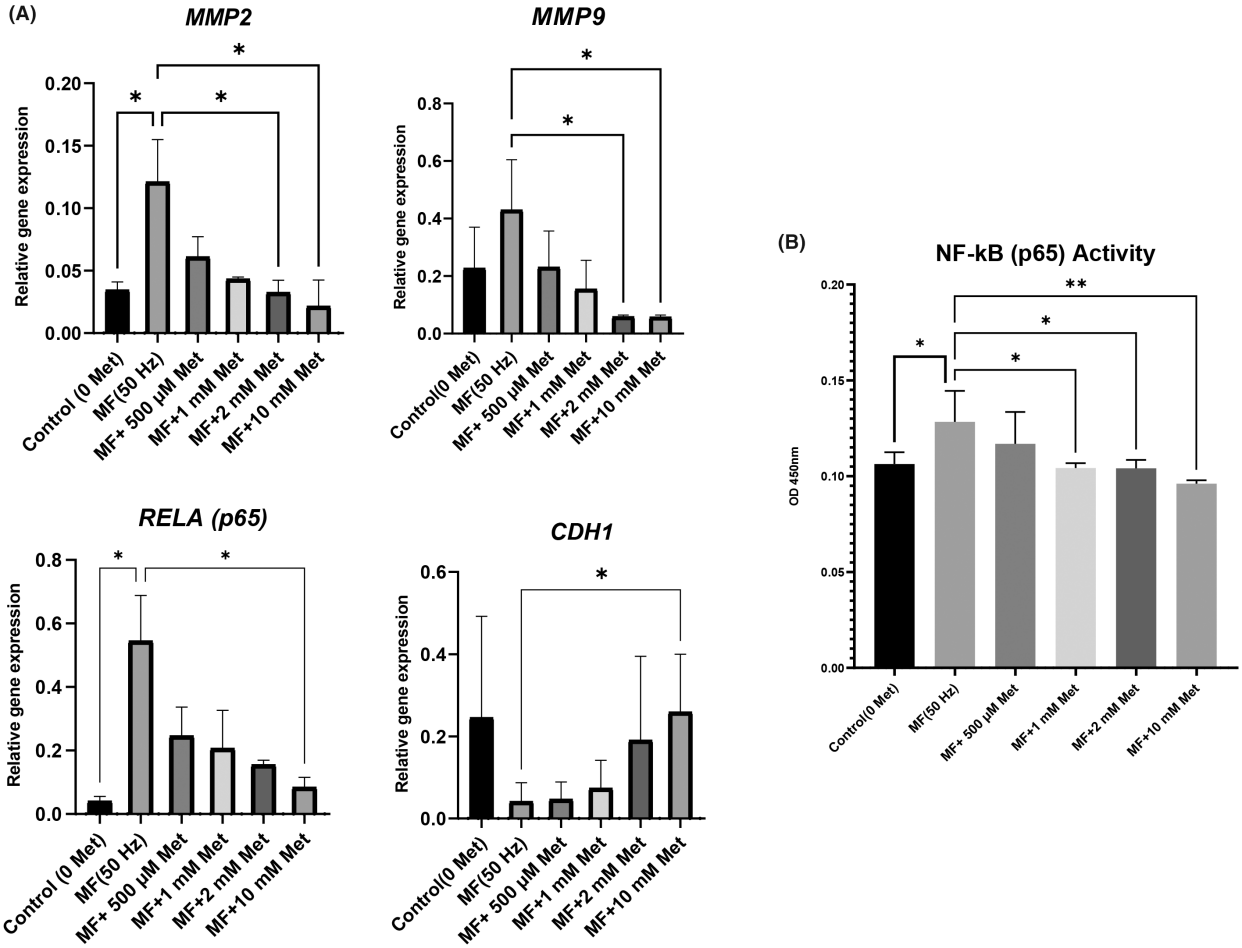
(A) Effects of metformin (500 μM, 1 mM, 2 mM and 10 mM) treatment on RELA /p65, CDH1, MMP2 and MMP9 expression levels in aged mouse fibroblast exposed to 50 Hz MF. *p < 0.05. Data were expressed as the mean ± SD from five independent experiments. (B) Effects of metformin (500 μM, 1 mM, 2 mM and 10 mM) treatment on NF‐kB (p65) DNA binding activity in aged mouse fibroblast exposed to 50 Hz MF. *p < 0.05, **p < 0.01. Data were expressed as the mean ± SD from five independent experiments.

We employed to quantify the mRNA expression levels of *MMP2/9*, *RELA* and *CDH1* genes of the aged NIH/3T3 cells exposed to 50 Hz MF by qRT–PCR. According to our findings, *MMP2/9* expression significantly increased under 50 Hz MF, compared to the control group (*p* < 0.05) (Figure 4A). However, the CDH1 expression was downregulated under Met‐free 50 Hz MF group. However, CDH1 expression was dramatically upregulated at a high Met dose (10 mM) compared to the Met‐free 50 Hz MF group (*p* < 0.05). Besides, MMP 2/9 mRNA significantly decreased at two different doses of Met administration (2 mM and 10 mM, respectively) under 50 Hz MF, compared to the Met‐free 50 Hz MF group (*p* < 0.05).

The RELA/p65 expression significantly elevated in the Met‐free 50 Hz MF group compared to the control group (*p* < 0.05). Conversely, at 50 Hz MF, a high dose of Met (10 mM) significantly decreased RELA/p65 expression (*p* < 0.05) (Figure 4A).

The findings indicate that 10 mM Met, in particular, downregulates the expression of the RELA/p65 and MMP2/9 genes, while upregulating the expression of CDH1.

### 3.5 Inhibition of NF‐kB/p65 DNA binding activity by Met in aged NIH/3T3 cells exposed to 50 Hz MF

ELISA was used to evaluate the DNA‐binding activity of NF‐kB/p65. The activity of NF‐kB/p65 was significantly elevated in NIH/3T3 aged mice fibroblasts after 1 h of exposure to 50 Hz MF (*p* < 0.05). The results suggested that Met treatment (500 M, 1 mM, 2 mM and 10 mM) suppressed NF‐kB/p65 activity in aged mouse fibroblasts exposed to 50 Hz MF at 1 h (Figure 4B). Met effectively suppresses the DNA‐binding activity of NF‐kB/p65 in aged NIH/3T3 cells after 50 Hz MF exposure, with the most significant suppression observed at 10 mM Met concentration, suggesting a potential mechanism for reducing detrimental effects of 50 Hz MF by regulating NF‐κB activity.

## 4 DISCUSSION

The aging process, which is a well‐established risk factor for various disorders, such as cancer, is associated with a homeostatic imbalance, including genotoxic and oxidative stress.^36^ Some cell‐based and epidemiological studies have suggested that 50–60 Hz MFs‐induced oxidative and genotoxic stress increases the risk of developing cancer.^2^, ^3^, ^4^, ^5^, ^6^ Carcinogenesis is a multi‐stage process that gradually transforms normal cells into malignant cells. These stages involve the maintenance of proliferative signalling, the evasion of growth suppressors, the resistance to cell death, the attainment of replicative immortality, the activation of invasion and metastasis and the induction of angiogenesis. These hallmarks collectively define the critical processes that drive the progression from normal cells to cancer.^37^ In our previous research, we demonstrated that the expression of MMPs in both primary breast cancer cells and the MCF‐7 cell line is regulated by the transcription factor NF‐κB. NF‐κB is a pivotal factor in carcinogenesis, playing essential roles not only in inflammation but also in key processes such as metastasis, cell proliferation, cell viability and invasion.^25^, ^38^

Excessive activation of the NF‐κB signalling pathway is a prevalent feature in diverse tumour tissues. Consequently, studies focused on targeting this pathway for cancer therapy have become a significant area of research. Recently, NF‐κB inhibitors have been widely employed to reduce cancer metastasis by suppressing the expression of several prometastatic genes. Moreover, these drugs may also inhibit the metastasis of other cancer types, including prostate, brain, melanoma, ovarian and pancreatic cancers.^39^ Met, an NF‐κB inhibitor that has demonstrated clinical safety for the treatment of type 2 diabetes mellitus (T2DM), has garnered attention from oncologists as a potential cancer therapeutic. This antioxidant appears to be a promising anti‐cancer and anti‐aging agent.^22^, ^32^, ^33^

Excessive proliferation of fibroblasts is known to be a critical factor in carcinogenesis; however, the molecular machinery of how 50 Hz MF impairs cell growth and how Met fixes such harmful conditions have not yet been elucidated.^40^ In this study, we demonstrated that 50 Hz MF significantly increased the cell proliferation and viability of aged NIH/3T3 mouse fibroblasts. Some researchers have shown an increase in the proliferation of both cancer cells and normal cells after exposure to ELF‐MF. In line with our findings, previous studies have reported that exposure to 0.5–5 mT MF promotes cell growth in various cell lines, including prostate cancer cell lines, human epidermal stem cells, WI‐38 diploid fibroblasts, rat‐1 fibroblasts and HL‐60 leukaemia cells.^41^, ^42^, ^43^ In this context, Wolf and collaborators showed that exposure of HL‐60 leukaemia cells and rat fibroblasts to 50 Hz (0.5–1.0 mT) ELF‐MF affected cell proliferation and DNA damage through the action of free radical species.^43^ According to our findings, this 50 Hz MF‐mediated promotion of cell proliferation was under the control of 24 h Met administration in a concentration‐dependent manner. Moreover, the 50 Hz MF significantly increased the viability of the aged NIH/3T3 fibroblast, whereas the Met administration, in a dose‐dependent manner, restored the number of viable cells. Many studies have suggested that Met may limit the growth of many tumour cells.^25^, ^32^, ^38^ Our previous in vitro study using primary breast cancer cells (PBCCs) showed that Met can inhibit cell proliferation through an AMPK‐alpha independent mechanism in breast cancer cells.^25^

Numerous age‐related diseases, including cancer, have been linked to the overactivation of NF‐κB, a crucial transcription factor.^22^, ^25^, ^38^ Our previous research has emphasized the pivotal role of NF‐κB in both skin aging and breast cancer.^23^, ^24^, ^25^ To explore the potential impact of ELF‐MF on NF‐κB activity in aging cells, we investigated its DNA‐binding capacity in aged NIH/3T3 cells. Our findings revealed that exposure to 50 Hz ELF‐MF significantly increased NF‐κB DNA‐binding activity in these cells. Following the present results, Wolf et al., have suggested that 50 Hz low‐frequency MF triggered the DNA‐binding activity of NF‐kB (p65) in Rat‐1 fibroblasts, WI‐38 diploid fibroblasts and HL‐60 leukaemia cells.^43^ However, the administration of Met, an NF‐κB inhibitor, effectively counteracted this effect, suggesting its potential therapeutic benefits as an anti‐aging and anti‐cancer agent.

Accumulating evidence indicates that NF‐kB signalling is required for EMT in cancerogenesis.^27^, ^44^ Cancer cells undergo EMT, a process characterized by the loss of proteins that support cell–cell contact, such as E‐cadherin. This is accompanied by the acquisition of mesenchymal markers, including vimentin and metalloproteinases such as MMP‐2 and MMP‐9, which enhance cell motility and invasion. Furthermore, NF‐κB has been implicated in the regulation of EMT marker genes, including *CDH1*, *MMP2* and *MMP9*.^27^, ^28^ We showed that a high dose of Met administration (10 mM) for a 24‐h period downregulated RELA and MMP2/9 expressions, while upregulating CDH1 expression via NF‐kB activity in the 50 Hz MF exposed aged NIH/3T3 cells. A significant reduction in MMP‐2/9 expression was observed at the 10 mM Met dose, as *indicated* by both immunocytochemistry and real‐time *PCR*. Previous research within melanocytic lineages has demonstrated Met's ability to inhibit EMT, upregulate E‐cadherin expression, and downregulate N‐cadherin at both the gene and protein levels.^45^ In a migration study involving the MDA‐MB‐231 cell line, Met at a concentration of 20 mM was found to suppress the expression of MMP‐2 and MMP‐9.^46^ Our earlier studies align with these findings, revealing a similar decrease in MMP‐2 and MMP‐9 expression at high doses of Met in MCF‐7 cells. These collective results are consistent with previous reports conducted by Sharma&Kumar (2018) and Besli et al. (2020).^38^, ^45^ It is encouraging to compare our findings with those found by Patruno et al., who found induced expression of MMP‐9 in Hacat keratinocyte cells at 50 Hz MF exposure.^47^ In another paper, Zhu et al., reported that 50 Hz MF exposure time‐dependently promoted MMP‐2 expression in cultured human fetal scleral fibroblasts, as indicated by both Western blot and RT‐PCR analysis.^48^ Therefore, this study suggests that Met may counteract the detrimental effects of 50 Hz MF on aged NIH/3T3 fibroblasts by regulating NF‐κB activity and the expression of, *CDH1*, *MMP2/9* and *RELA*.

## 5 CONCLUSION

**FIGURE 5:**
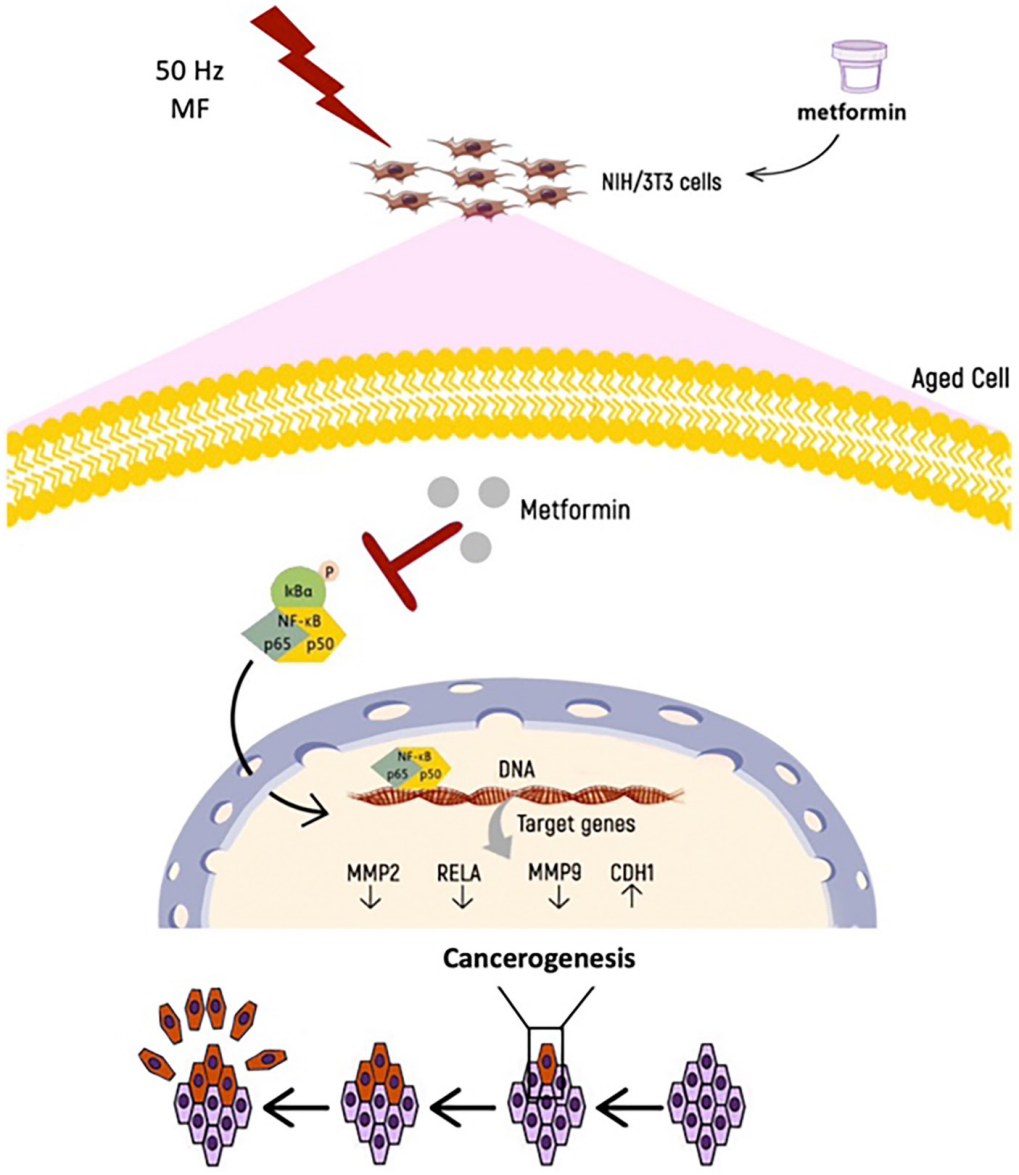
A schematic diagram showing the mechanism by which in vitro protective effects of metformin against carcinogenesis are potentially induced by a 50 Hz magnetic field in aged mouse fibroblasts.

In conclusion, our study suggests that a 50 Hz uniform MF promotes the aged mouse fibroblast cell viability, proliferation and expression of MMP genes through an elevated NF‐kB activity while decreasing the expression of E‐cadherin. Met may mitigate the detrimental effects triggered by this 50 Hz MF on the aged mouse fibroblasts by modulating the EMT entities and NF‐kB, which possibly plays a crucial role in the development of 50 Hz MF‐induced carcinogenesis and hence might be a future therapeutic candidate for treating MF‐induced cancer. The findings presented in this study demonstrate the potential impact of ELF‐EMF on the carcinogenic process, highlighting the importance of further investigating its role in this complex phenomenon. Additionally, our results suggest that Met, an NF‐κB inhibitor, may mitigate the adverse effects of 50 Hz MF on aged mouse fibroblasts by regulating the expression of MMP 2/9 and E‐cadherin (Figure 5). Both in vitro and in vivo evidence supports a link between exposure to ELF‐EMF and DNA strand breaks. However, conflicting findings regarding the therapeutic effects of these fields underscore the need for a more comprehensive understanding of their biological mechanisms. This study has several limitations. First, the results are limited to a single cell line level, so to understand the overall biological effects of ELF‐EMFs, the effect of a uniform ELF‐EMF should be further investigated in various human cell types and in vivo models. Evaluation of additional primary cell lines, along with a wider examination of aging and cancer markers, is necessary. Second, the existing literature on the impact of low‐frequency electroMFs presents conflicting results. Therefore, the generalizability of our preliminary findings is limited and requires cautious interpretation.

## AUTHOR CONTRIBUTIONS

**Tugba Soydas:** Conceptualization (lead); data curation (equal); formal analysis (lead); funding acquisition (lead); investigation (equal); methodology (equal); project administration (equal); writing – original draft (lead); writing – review and editing (lead). **Guven Yenmis:** Conceptualization (supporting); formal analysis (supporting); funding acquisition (supporting); investigation (supporting); methodology (supporting); project administration (supporting); writing – original draft (supporting); writing – review and editing (supporting). **Matem Tuncdemir:** Conceptualization (supporting); formal analysis (supporting); funding acquisition (equal); investigation (supporting); methodology (supporting); writing – original draft (supporting); writing – review and editing (supporting). **Mustafa Tunaya Kalkan:** Formal analysis (supporting); investigation (supporting); methodology (supporting). **Elif Yaprak Sarac:** Investigation (supporting); methodology (supporting); writing – original draft (supporting); writing – review and editing (supporting). **Ayhan Bilir:** Investigation (supporting); methodology (supporting); writing – original draft (supporting); writing – review and editing (supporting). **Gonul Kanigur Sultuybek:** Conceptualization (supporting); formal analysis (supporting); investigation (supporting); methodology (supporting); project administration (supporting); writing – original draft (supporting); writing – review and editing (supporting).

## CONFLICT OF INTEREST STATEMENT

The authors declare no conflicts of interest.

## Data Availability

The supplementary findings of the study are available upon request.
